# Mania With Psychotic Symptoms a Rare Clinical Presentation of Fahr's Disease- a Case Report

**DOI:** 10.1192/bjo.2022.361

**Published:** 2022-06-20

**Authors:** Joana Marques Pinto, José Diogo Andrade, Joana Andrade, Vera Martins

**Affiliations:** CHUC, Coimbra, Portugal

## Abstract

**Aims:**

Fahr's disease is a rare neurodegenerative disease with radiological findings of symmetrical and bilateral idiopathic calcifications of the cerebellum, periventricular white matter, and basal ganglia, characterized by the presence of neuropsychiatric symptoms.

**Methods:**

We report a case of a 46-year-old male who presented with psychomotor agitation, aggression, restlessness, irritability, decrease need for sleep and psychotic symptoms including grandeur and mystical delusions. He had a previous history of an admission 4 years prior with a similar presentation. Computed tomographic scan of the patient demonstrated a bilateral calcification of globus pallidus. Laboratory investigation was unremarkable. Due to agitation, the patient started treatment with Haloperidol 10 mg and Levomepromazine 25 mg presenting resulting in important extra-pyramidal symptoms (EPS), namely marked motor rigidity. Subsequently, a switch was made to Olanzapine 5 mg with persistence of clinically significant EPS. A final switch was made to Aripiprazol 15 mg (gradually titrated) and a mood stabilizer was started (Sodium Valproate), with full clinical remission within a month and no signs of EPS.

**Results:**

The age of onset of manic symptoms in this patient is not suggestive of bipolar disorder (average age onset 25). On the other hand, Fahr's disease usually presents within the 4th and 5th decade of life. The clinical presentation usually involves motor symptoms (movement disorder and Parkinson like symptoms) and dementia, but purely psychiatric presentations have been described. The localization of calcifications also seems to have a clinical correlation, as Pallidal calcifications as the ones identified in our patient have been associated with manic symptoms. Idiopathic forms in which no metabolic or other underlying causes are identified, treatment is usually symptomatic, but one has to be cautious because these patients have an increased sensitivity to neuroleptics and can thus easily develop EPS.

**Conclusion:**

Psychiatrists should consider Fahr's disease as a differential diagnosis in a manic episode, especially with a late age of onset, which is not suggestive of a bipolar disorder. This case also further emphasizes the importance of neuro-imaging in psychiatry and underlines the importance of a careful treatment approach in this type of patients because of an higher risk of developing EPS.

